# Integrating gender equity and social inclusion into forecasting and supply planning (FSP): a policy commentary

**DOI:** 10.1093/inthealth/ihag020

**Published:** 2026-03-11

**Authors:** Taroub Harb Faramand, Sara Pappa, Sara Jane Renfroe, Laila Akhlaghi

**Affiliations:** WI-HER, Vienna, VA 22182, USA; Ponte Vedra Beach, FL 32082, USA; Falls Church, VA 20598, USA; WI-HER, Vienna, VA 22182, USA; San Miguel Chapultepec, Mexico City 11850, Mexico; Arlington, VA 22202, USA

## Abstract

This policy commentary argues that forecasting and supply planning (FSP) for immunization systems must integrate gender equity and social inclusion (GESI) to bridge the gap between vaccine supply and actual utilization. While FSP traditionally focuses on supply-side efficiency, this narrow approach risks perpetuating inequities by leaving systematically excluded populations invisible in data and planning processes. Drawing on experience from the Immunization Collaborative Supply Planning Strengthening project, we demonstrate how contextual factors including gender dynamics, mobility patterns, seasonality and conflict shape vaccination demand among marginalized communities. The commentary outlines key practice implications, including strengthening supply–demand collaboration, embedding GESI expertise into FSP teams and improving data disaggregation. We conclude that this integration is essential to transforming forecasting into an inclusive mechanism that ensures equitable immunization access.

## Introduction: Why This Matters

Forecasting and supply planning (FSP) sits at the heart of reliable immunization systems, determining whether essential childhood vaccines are available and where and when they are needed. Effective FSP ensures that supply aligns with demand, stockouts are minimized and wastage is reduced. However, when forecasting is narrowly focused on the supply side, it risks missing a critical dimension: demand and utilization.

Improving supply without addressing the demand side can inadvertently lead to waste. Providers who concentrate exclusively on forecasting based on usage or stock data may overestimate or underestimate needs if they fail to account for changes in behavioural, social or contextual barriers that limit utilization. While baseline usage patterns are captured in historical data, it is the shifts and disruptions in these patterns that require active monitoring and adjustment. Therefore, FSP should go hand in hand with demand generation, ensuring that supply-side efficiency is complemented by efforts to increase awareness, accessibility and uptake of immunization services.

For the purposes of this commentary, three terms are used distinctly but relationally. Demand refers to the expressed or latent need for immunization services within a population, shaped by social, economic, gender and contextual factors. Utilization refers to the actual uptake of vaccines—whether services are accessed and delivered. FSP translates anticipated demand into procurement, allocation and distribution decisions. When these concepts are conflated, forecasting risks mistaking historical utilization for true demand, thereby reproducing patterns of exclusion rather than correcting them.

‘A chain is only as strong as its weakest link, and demand forecasting is one of the weakest links of health supply chains’.^1^

Just as sales and planning teams must understand marketing strategies to forecast accurately, supply chain forecasters must be aware of demand-side interventions and barriers to adjust their projections accordingly. To date, global health FSP has largely been approached as a technical exercise in efficiency and optimization, particularly in crisis contexts. Forecasts rely heavily on demographic estimates and administrative health data, combined with logistics algorithms, to project demand and guide procurement. While these models are increasingly sophisticated, they tend to treat data as static inputs rather than reflections of dynamic social realities.

The missing element is equity and inclusion. Systems have not been adequately designed to identify who is systematically left out or why. Beyond the ‘known unknowns’—populations we recognize as underserved—forecasting systems fail to illuminate the ‘unknown unknowns’—communities and barriers that remain invisible in current data structures and demand assumptions.

Integrating gender equity and inclusion (GESI) into FSP does not mean adding demographic disaggregation as an afterthought, nor does it mean forecasting separately for women or marginalized groups. Rather, it means systematically incorporating social and gendered drivers of access, mobility and decision-making into how demand is interpreted, assumptions are set and forecasts are adjusted over time. In this sense, GESI integration is not an equity overlay on forecasting, it is a corrective to the structural blind spots embedded in conventional forecasting models, which can be a tipping point for the program.

A reimagined FSP process must explicitly ask: Who is not visible in these data? Whose needs are underrepresented? Who participates in these decisions that may bring the necessary insight? Such questions must be institutionalized within forecasting frameworks, supported by flexible mechanisms to identify, validate and adapt for underrepresented populations over time.

Vulnerable populations such as women in marginalized communities, children in nomadic or displaced households, people with disabilities and residents of conflict- or climate-affected areas are often excluded from administrative datasets. These gaps reflect both data system limitations and utilization barriers. For instance, weak vital registration systems and inaccurate birth or death records can distort population denominators, leading to underestimation of true demand.

The Immunization Agenda 2030 emphasizes equity and calls for strategies that reach zero-dose and underimmunized children by addressing barriers linked to poverty, geography, culture and gender. However, global strategies must go further to ensure that equity frameworks translate into actionable strategies that enhance utilization, not just supply.

When exclusion is baked into data, and data drives forecasts, inequity becomes self-reinforcing. Forecasts that appear technically sound can nonetheless fail operationally, because they are calibrated to utilization patterns shaped by exclusion rather than to underlying population needs.

In short, forecasting is not just a supply function, it is a bridge between availability and access. Without connecting the supply and demand sides, even well-forecasted vaccines risk remaining unbought, unprocured, unsourced and ultimately unused, exacerbating inequity and inefficiency.

## Context: The Challenge of Equity in FSP

The challenge of equity in forecasting and supply planning is not only moral but analytical: when social exclusion shapes who appears in utilization data, forecasts based on those data systematically misrepresent true demand.

Demand is profoundly shaped by gender, mobility, geography and poverty. These socio-economic factors shape who seeks vaccines, who can access them and where gaps exist. Zero-dose children tend to cluster in marginalized communities—remote rural villages, fragile settings, urban slums and displacement camps. Yet these are not the only groups at risk. Migrant workers, informal settlers, nomadic groups, people with disabilities and minority populations also face systemic barriers to immunization access.

These inequities distort both data accuracy and forecasting reliability. Models grounded solely in historical consumption risk perpetuating past exclusion: if a group has been consistently undercounted, its demand remains invisible. Provider awareness and capacity are often limited. Supply-chain professionals focus primarily on logistics and utilization data without adequate understanding of social determinants influencing demand. Building their capacity to interpret data through an equity lens and fostering collaboration between supply chain managers and service delivery teams is critical.

Data reliability is another major constraint. Weak civil registration systems, incomplete birth and death data and irregular household surveys hinder accurate denominator estimates. Without integrating these data with local-level feedback on service utilization, forecasts risk being both numerically inaccurate and socially blind.

Leadership and gender inclusion also shape forecasting outcomes. True gender-responsive leadership entails more than appointing women to roles, it involves embedding GESI expertise in the review of data and decision-making processes and ensuring that leaders, regardless of their gender, possess the capacity to identify invisible populations and respond to equity challenges.

## Approach: How We Are Testing GESI Integration

Through the Immunization Collaborative Supply Planning Strengthening (ICSPS) project, supported by the Gates Foundation, WI-HER has operationalized ways to link supply planning with gender and social inclusion data. This work reframes GESI not as a synonym for women and girls, but as an analytic lens to understand inequities that affect all populations—those defined by gender, geography, mobility, disability or social norms.

The FSP Maturity Assessment Tool was adapted under the project to include measures, along with other supply chain factors, of inclusiveness by capturing gender representation, team diversity, GESI expertise and mechanisms for integrating equity into planning cycles. Data triangulation is another method to integrate GESI into the forecasting process. For example, comparing utilization data per segment of the population represented in demographic data, across jurisdictions, could help to identify pockets of unmet need. Data triangulation also engages frontline health workers and local authorities to interpret demand trends beyond what administrative data reveal.

For example, Nigeria is currently testing how factoring in women’s time use, seasonal migration and conflict-driven mobility can make projections more realistic and adaptive. This approach encourages collaboration between supply chain managers and service delivery teams, ensuring that improvements in supply directly enhance utilization, and vice versa.

Taken together, these approaches reposition forecasting as a learning system rather than a static projection exercise. GESI integration enables planners to test assumptions, interrogate anomalies in utilization data and adapt forecasts in response to changing social conditions rather than treating variance as an error.

## Case Insights from Early Country Experience

Following an ICSPS Technical Summit in Nairobi (June 2024), WI-HER initiated a deep dive with Nigeria to move from GESI assessment to operationalization. Over the past two years, the country has been implementing its ‘Big Catch Up’ program to better reach zero-dose children; this has shed light on the need for further development of a GESI-responsive supply chain to increase access for hard-to-reach communities. Thus WI-HER adapted a pipeline monitoring worksheet to incorporate GESI-focused assumptions, including seasonality, birth patterns, women’s time use during planting and harvesting seasons, pastoralist migration and conflict cycles.

Reflection sessions with Nigerian participants revealed how these factors shape demand. Agricultural seasons correlate with decreases in immunization attendance, and conflict-displaced populations exhibit sharp fluctuations requiring flexible planning. While the 2026 forecast proceeded using traditional methods, these insights will inform equity adjustments in the 2027 forecast workshop (second quarter of 2026), where GESI considerations will be integrated into actual forecast numbers.

At this point, Nigeria is progressing toward implementing this updated worksheet in its FSP in order to systematically incorporate variables such as seasonal migration and conflict-driven population mobility into annual state-level supply planning. For states affected by these dynamics, demand adjustments and distribution strategies will be made to reflect temporal population shifts, access and utilization constraints, with the objective of optimizing product availability, minimizing stock imbalances and improving service continuity throughout the planning period.

## Practice Implications for the Wider Field

Before turning to recommendations, it is important to clarify why these implications matter. Supply chain forecasting is not only about technical efficiency, it is about ensuring equitable access and system responsiveness. Addressing these dual goals requires structural and cultural change within FSP systems.

The implications below follow directly from the central argument of this commentary: that forecasting must move beyond static consumption or demographic models toward adaptive, equity-responsive systems. Each recommendation addresses a specific failure point identified earlier—whether in data interpretation, team composition or the disconnect between supply planning and service delivery.

- Align supply and demand through stronger collaboration between supply chain and service delivery teams.
- Institutionalize GESI expertise within FSP teams so that equity considerations actively shape assumptions, scenario building and forecast adjustments.
- Strengthen provider capacity for adaptive and equity-informed planning.
- Enhance data systems through improved disaggregation by sex, age, disability, mobility and other determinants affecting access and utilization, while identifying and addressing biases in data collection and analysis, including those introduced by artificial intelligence–driven forecasting tools.
- Promote inclusive leadership that values both representation and expertise.
- Foster local co-creation by engaging communities to validate assumptions.
- Reframe wastage as an equity issue, encouraging vaccination of every eligible child while improving planning to minimize unnecessary loss.

## Conclusions

When forecasting functions as a true bridge between supply and utilization rather than a one-way projection from past consumption (or known/or identified demographic data) it becomes a tool not only for efficiency but for accountability, adaptability and equity.

## Data Availability

The data underlying this article will be shared upon reasonable request to the corresponding author.
